# Secular Trends in the Prevalence of Small Vulnerable Newborns in Japan, 1997–2021

**DOI:** 10.2188/jea.JE20240447

**Published:** 2026-03-05

**Authors:** Keisuke Yoshii, Hibiki Doi, Mizuho Igarashi, Kohei Ogawa, Keiko Matsubara, Tetsuya Isayama, Kenichi Kashimada, Naho Morisaki

**Affiliations:** 1 Division of Endocrinology and Metabolism, National Center for Child Health and Development, Tokyo, Japan; 2 Department of Pediatrics, The University of Tokyo, Tokyo, Japan; 3 Center for Maternal-Fetal, Neonatal and Reproductive Medicine, National Center for Child Health and Development, Tokyo, Japan; 4 Division of Diversity Research, National Research Institute for Child Health and Development, Tokyo, Japan; 5 Division of Neonatology, National Center for Child Health and Development, Tokyo, Japan; 6 Department of Social Medicine, National Center for Child Health and Development, Tokyo, Japan

**Keywords:** small vulnerable newborn, small for gestational age, preterm, secular trend, Japan

## Abstract

**Background:**

In 2023, a collaborative United Nations Children’s Fund–World Health Organization group introduced the concept of small vulnerable newborns (SVNs) to improve the identification of newborns at increased risk of adverse outcomes and to guide more effective preventive strategies. However, global data on the prevalence of SVNs remains scarce. This study aimed to examine secular trends in the prevalence of SVNs and their three subgroups, namely term small for gestational age (SGA), preterm SGA, and preterm non-SGA, in the Japanese population.

**Methods:**

We analyzed data from vital statistics including livebirths and stillbirths between 1997 and 2021. Secular trends in the prevalence of SVNs and their subgroups were assessed. In addition, we conducted regional analyses to explore associations with the distribution of medical resources.

**Results:**

A total of 26,172,760 newborns were included. The overall prevalence of SVNs was 7.8% in 1997, peaked at 8.7% in 2005, and declined to 7.7% by 2021. This decline was primarily attributed to a reduction in term SGA births. In contrast, the prevalence of preterm SGA and preterm non-SGA remained largely unchanged. In 2021, the prevalence of term SGA, preterm SGA, and preterm non-SGA was 2.0%, 0.44%, and 5.3%, respectively. No significant association was found between the regional variation in the prevalence of SVNs and the distribution of medical resources.

**Conclusion:**

The prevalence of SVNs in Japan has declined since 2005, mainly due to reduced term SGA births. Persistent rates of preterm subgroups highlight the ongoing burden of prematurity, underscoring the need for targeted strategies to improve neonatal outcomes.

## INTRODUCTION

Birth size is a well-established determinant of neonatal mortality and morbidity. Low birth weight (LBW), a birth weight of less than 2,500 g, has traditionally been used as a global metric. However, LBW is a heterogeneous category that encompasses both preterm birth and fetal growth restriction (FGR), two conditions with distinct etiologies, risk factors, and prevention strategies. Preterm birth was defined as delivery before 37 weeks of gestation in 1977 by the World Health Organization (WHO).^1^ The concept of small for gestational age (SGA), closely related to FGR, was introduced in 1995.^2^ SGA integrates birth weight, birth length, gestational age, and population-based references. In general, SGA is determined when both a neonate’s birth weight and birth length are below the 10^th^ percentile for their gestational age or when a birth weight and/or a birth length is more than two standard deviations below the mean for their respective gestational age.^3^^,^^4^

Despite longstanding global recognition of the importance of reducing LBW and SGA to improve neonatal and child health outcomes, progress in their reduction has been slower than expected.^2^ A United Nations Children’s Fund (UNICEF)–WHO collaboration team, publishing in *The Lancet* in 2023, introduced the Small Vulnerable Newborns (SVNs) framework, an umbrella category that includes LBW, SGA, and preterm birth.^2^^,^^5^^–^^7^ In practice, the SVN category primarily centers around SGA and preterm rather than LBW. This shift underscores the primary role of SGA and preterm birth in neonatal vulnerability. The 2,500 g threshold for LBW, established nearly a century ago, remains widely used but does not fully capture the complexity of neonatal vulnerability. The SVN framework aims to improve problem identification and optimize prevention efforts by prioritizing interventions targeting preterm and SGA births.

Global priorities in perinatal care differ by context. In low- and middle-income countries, the concern is improving neonatal survival rates in the face of high mortality and limited health resources.^2^ In contrast, high-income countries face the challenge of mitigating the long-term developmental and metabolic consequences of children with FGR and prematurity.^2^^,^^8^ In addition, disparities in maternal and newborn health persist even in high-resource settings, driven by socioeconomic inequalities, such as access to prenatal and perinatal care and environmental exposures.^9^^,^^10^ The SVN framework is essential to provide a comprehensive and globally relevant approach to addressing neonatal vulnerability across diverse socioeconomic contexts. However, global epidemiological data on SVNs remain limited, primarily due to the lack of comprehensive data on SGA prevalence. Recognizing the critical role of accurate SVN data in improving neonatal outcomes, international organizations have emphasized the need for enhanced data collection and reporting.^5^ For meaningful international comparisons, it is crucial that Japan, one of the high-income countries with the lowest neonatal mortalities and the highest standard of obstetric care, contributes reliable and comprehensive data.

While studies from Japan have reported increased risks of developmental delays and behavioral problems among children born SGA and/or prematurely,^11^^–^^15^ other studies have identified modifiable factors associated with LBW and SGA.^16^^–^^18^ These include smoking, underweight status before pregnancy, and insufficient gestational weight gain. In response, public health efforts have focused on promoting smoking cessation among women of reproductive age and their partners.^19^ Japanese obstetric guidelines revised the recommendation not to impose strict weight gain restrictions in pregnant women, after highlighting the risks of staying slim during pregnancy for Japanese women in 2018.^20^^–^^22^ Furthermore, preconception care initiatives focus on encouraging women and couples to optimize their health in preparation for pregnancies, aiming to improve pregnancy outcomes.^23^^–^^26^

The national initiative “Healthy Parents and Children 21”, led by Japanese governmental agencies, has pointed out regional disparities in perinatal and infant mortality rates at the prefectural level, highlighting the need for targeted public health interventions.^27^ However, given that Japan maintains one of the lowest perinatal mortality rates globally, such indicators might lack sufficient sensitivity to capture regional disparities in maternal and child health. Examining the prevalence and distribution of SVNs may offer a more accurate measure, facilitating the identification of regional health inequalities and informing the more effective allocation of healthcare resources.

Japan’s system of vital statistics, founded in 1899 under the Family Registry Law and the Provisions Regarding Notification of Stillbirths, is administered by the Ministry of Health, Labour and Welfare.^28^ Upon submission of notification forms to municipal authorities, copies are forwarded to the ministry, where they are compiled into the national vital statistics database. This system is based on five primary registration records: births, stillbirths, deaths, marriages, and divorces. Birth and stillbirth registrations capture key perinatal parameters, including gestational age, birth weight, birth length, singleton or multiple birth status, parity, and nationality. Given that stillbirths are associated with severe FGR, the UNICEF–WHO group has emphasized the need to incorporate stillbirth data to avoid underestimating the burden of SVNs.

To address the gap in perinatal health surveillance and to capture the overall trends in SVNs as potential outcomes of population-level health interventions, this study examines secular trends in the prevalence and regional distribution of SVNs in Japan using vital statistics between 1997 and 2021. The findings aim to facilitate both international and domestic comparisons.

## METHODS

### Study population

The study protocol was approved by the Ethical Committee of the National Center for Child Health and Development on September 19, 2023 (approval number: 2023-093). Data access to vital statistics is approved following a review process to ensure compliance with ethical and legal standards. We obtained approval to analyze the data for this study on May 17, 2023.

This study was limited to the surveys from 1997 to 2021 for three reasons. First, individual data before 1979 were not accessible at study initiation. Second, vital statistics recorded between 1979 and 1994 did not specify the gestational age with the required precision because they recorded only the number of weeks, not the exact days (eg, 40 weeks but not 40+0 weeks). Third, the reliability of gestational age data was compromised for 1995 and 1996 in the birth registration, as detailed in the supplementary materials (eTable 1).

### Inclusion and exclusion criteria and definition of SGA

This study included birth and stillbirth registrations from 1997 to 2021. The exclusion criteria were applied to babies if both parents were non-Japanese nationals. In this study, SGA was defined as babies with both birth weight and length below the 10^th^ percentile (corresponding to a z-score of −1.28). The z-scores for individual birth weights and lengths were calculated using the Japanese reference values established in 2014.^29^ Preterm babies were defined as those born before 37 weeks of gestation. The Japanese reference values apply to babies born between 22 and 41 weeks of gestation; therefore, babies born before 22+0 weeks or after 41+6 weeks were excluded from the analysis.

### Statistical analysis

First, we examined secular trends in the prevalence of LBW and SVNs, with a particular focus on the three subgroups of SVN, namely, term SGA (neonates with SGA born between 37–41 weeks of gestation), preterm SGA (neonates with SGA born between 22–36 weeks of gestation), and preterm non-SGA (neonates without SGA born between 22–36 weeks of gestation). Second, to illustrate secular trends in the composition of the subgroups of SVN, we utilized a 100% stacked bar chart to depict the relative proportion of the three subgroups. Third, we analyzed regional variations in SVN prevalence at the prefectural level between 2017 and 2021 and examined its correlation with the distribution of medical resources. The analysis included population size and land area per perinatal medical center, defined as 395 tertiary medical institutions nationwide in 2017, each equipped with obstetrics and neonatology departments.

A complete-case analysis approach was employed. Statistical significance was set at *P* values <0.05, and all tests were two-tailed. All statistical analyses were conducted using STATA v14.2 (STATA Corp, College Station, TX, USA).

## RESULTS

**Figure 1.**
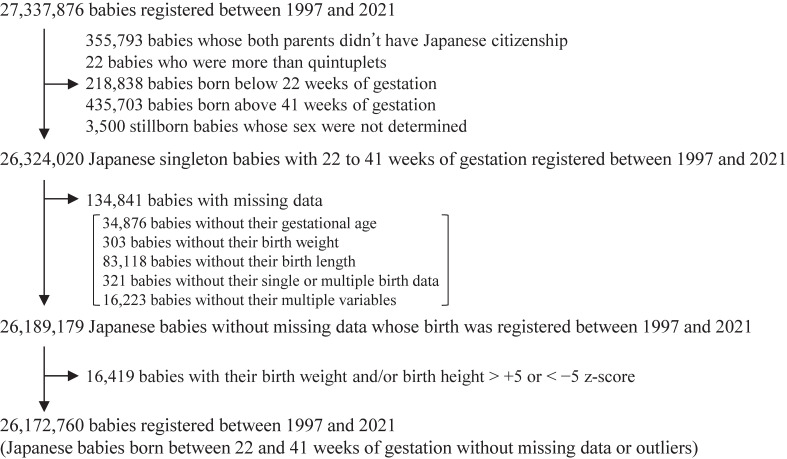
Population flowchart

Figure 1 presents a population flowchart. Between 1997 and 2021, a total of 27,337,876 livebirths and stillbirths were registered. We excluded 355,793 babies with non-Japanese parents, 22 babies who were more than quintuplets, 654,541 babies born before 22 weeks or after 41 weeks of gestation, 3,500 stillborn babies whose sex was not determined, 134,841 babies with missing data, and 16,419 babies identified as outliers. After these exclusions, this study analyzed 26,172,760 Japanese babies.

In 1997, the prevalence of LBW and SVN was 8.1% and 7.8%, respectively. The prevalence of LBW increased steadily, reaching a peak of 9.8% in 2007, followed by stabilization and a slight decline to 9.4% by 2021 (Table 1). In contrast, the prevalence of SVNs peaked earlier at 8.7% in 2005, followed by a decline to 7.7% in 2021. Among SVN subgroups, the values in 1997 were 2.6% for term SGA, 0.41% for preterm SGA, and 4.8% for preterm non-SGA. The prevalence of term SGA increased modestly to 2.9% by 2002 and then began to decrease. The prevalence of preterm SGA peaked at 0.54% in 2005, followed by a plateau and gradual decline. In contrast, the prevalence of preterm non-SGA continued to rise, reaching 5.4% by 2007, after which it plateaued. In 2021, the values of term SGA, preterm SGA, and preterm non-SGA were 2.0%, 0.44%, and 5.3%, respectively.

**Table 1.** Annual trends in the number and proportion of LBW, SVNs, and the three SVN subgroups from 1997 to 2021

Year	Total	LBW		SVNs		Preterm SGA		Preterm non-SGA		Term SGA	
	*N*	*N*	%	*N*	%	*N*	%	*N*	%	*N*	%
1997	1,175,440	95,392	8.12	91,823	7.81	4,857	0.41	56,141	4.78	30,825	2.62
1998	1,188,932	99,236	8.35	95,080	8.00	5,245	0.44	57,466	4.83	32,369	2.72
1999	1,165,472	100,805	8.65	94,851	8.14	5,310	0.46	57,154	4.90	32,387	2.78
2000	1,180,267	104,421	8.85	97,923	8.30	5,172	0.44	60,173	5.10	32,578	2.76
2001	1,161,510	104,427	8.99	96,458	8.30	5,571	0.48	58,220	5.01	32,667	2.81
2002	1,145,496	105,711	9.23	97,019	8.47	5,585	0.49	58,256	5.09	33,178	2.90
2003	1,116,528	103,649	9.28	94,709	8.48	5,649	0.51	57,926	5.19	31,134	2.79
2004	1,104,992	106,107	9.60	95,795	8.67	5,615	0.51	58,658	5.31	31,522	2.85
2005	1,057,982	102,319	9.67	92,121	8.71	5,712	0.54	55,822	5.28	30,587	2.89
2006	1,088,644	105,395	9.68	94,205	8.65	5,792	0.53	57,429	5.28	30,984	2.85
2007	1,086,407	105,992	9.76	94,388	8.69	5,775	0.53	58,180	5.36	30,433	2.80
2008	1,088,719	105,408	9.68	93,051	8.55	5,624	0.52	58,122	5.34	29,305	2.69
2009	1,067,843	103,543	9.70	90,199	8.45	5,511	0.52	56,283	5.27	28,405	2.66
2010	1,069,874	104,013	9.72	90,191	8.43	5,437	0.51	56,748	5.30	28,006	2.62
2011	1,049,231	101,055	9.63	87,450	8.33	5,328	0.51	55,589	5.30	26,533	2.53
2012	1,036,093	99,988	9.65	86,066	8.31	5,230	0.50	54,956	5.30	25,880	2.50
2013	1,028,395	99,179	9.64	85,078	8.27	5,228	0.51	54,538	5.30	25,312	2.46
2014	1,002,558	96,304	9.61	81,817	8.16	5,042	0.50	52,363	5.22	24,412	2.43
2015	1,004,641	95,740	9.53	80,771	8.04	4,924	0.49	51,679	5.14	24,168	2.41
2016	976,222	92,522	9.48	77,651	7.95	4,646	0.48	50,279	5.15	22,726	2.33
2017	944,978	89,662	9.49	75,067	7.94	4,463	0.47	49,319	5.22	21,285	2.25
2018	917,162	86,456	9.43	71,706	7.82	4,204	0.46	47,615	5.19	19,887	2.17
2019	864,603	81,714	9.45	67,454	7.80	3,952	0.46	44,802	5.18	18,700	2.16
2020	840,025	77,629	9.24	63,493	7.56	3,835	0.46	42,317	5.04	17,341	2.06
2021	810,746	76,179	9.40	62,501	7.71	3,596	0.44	42,836	5.28	16,069	1.98

LBW, low birth weight; SGA, small for gestational age; SVNs, small vulnerable newborns.

Note: This table includes both livebirths and stillbirths.

**Figure 2.**
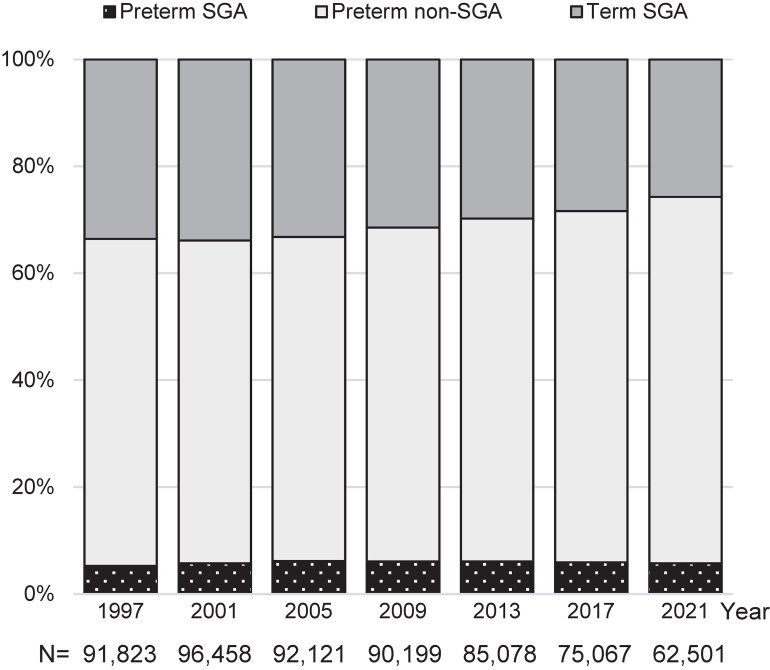
Secular trends in the distribution of the three SVN subgroups. SGA, small for gestational age; SVN, small vulnerable newborn.

Figure 2 displays a 100% stacked bar chart of SVN subgroup proportions. In 1997, term SGA accounted for 33.6%, preterm SGA for 5.3%, and preterm non-SGA for 61.1%. The relative proportion of term SGA revealed a declining trend to 25.7% in 2021. The relative proportion of preterm SGA showed a slight increase by 2005, followed by a modest decline to 5.7% in 2021. Preterm non-SGA consistently held the largest proportion over the years, and its relative proportion increased to 68.5% in 2021.

**Figure 3.**
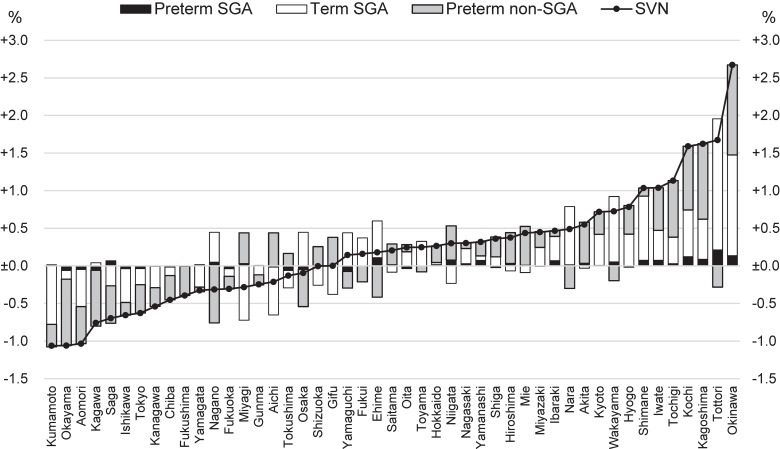
Differences in the prevalence of SVNs from 2017 to 2021 between each prefecture and the national average. SGA, small for gestational age; SVN, small vulnerable newborn.

Regional variation, which is the differences in SVN prevalence for 5 years from 2017 to 2021 between each prefecture and the national average, is illustrated in Figure 3. The differences were not significantly associated with medical resource distribution (*R*^2^ = 0.05, *P* = 0.149 for prefecture population size per a perinatal medical center; *R*^2^ = 0.03, *P* = 0.214 for prefecture land area per a perinatal medical center). eTable 2 provides data collected from national surveys in the regional analysis.

Table 2 shows the annual trends in the proportion of SVN by livebirths or stillbirths and singletons or multiple births. The SVN proportion among stillbirths remained consistently high (∼80%) throughout the study period, substantially exceeding that among livebirths. Similarly, the SVN proportion among multiple births was consistently higher than among singletons. Annual SVN prevalence of livebirths, singletons, and multiple births groups followed a similar trajectory, with an increase from 1997 to approximately 2005–2008, followed by a downward trend. Detailed data on the number and prevalence of the three SVN subgroups among livebirths or stillbirths (eTable 3) and among singleton or multiple births (eTable 4) are available in the supplementary materials.

**Table 2.** Number and proportion of SVNs among livebirths, stillbirths, singletons, and multiple births

Year	Livebirths			Stillbirths			Singletons			Multiple births		
	Total	SVNs		Total	SVNs		Total	SVNs		Total	SVNs	
	*N*	*N*	%	*N*	*N*	%	*N*	*N*	%	*N*	*N*	%
1997	1,170,319	87,669	7.49	5,121	4,154	81.1	1,154,043	79,978	6.93	21,397	11,845	55.4
1998	1,183,911	91,013	7.69	5,021	4,067	81.0	1,167,012	82,577	7.08	21,920	12,503	57.0
1999	1,160,607	90,870	7.83	4,865	3,981	81.8	1,143,153	81,820	7.16	22,319	13,031	58.4
2000	1,175,601	94,133	8.01	4,666	3,790	81.2	1,156,955	84,230	7.28	23,312	13,693	58.7
2001	1,156,972	92,720	8.01	4,538	3,738	82.4	1,138,599	82,702	7.26	22,911	13,756	60.0
2002	1,141,106	93,523	8.20	4,390	3,496	79.6	1,121,180	82,507	7.36	24,316	14,512	59.7
2003	1,112,424	91,431	8.22	4,104	3,278	79.9	1,091,984	79,830	7.31	24,544	14,879	60.6
2004	1,101,107	92,690	8.42	3,885	3,105	79.9	1,080,144	80,447	7.45	24,848	15,348	61.8
2005	1,054,379	89,264	8.47	3,603	2,857	79.3	1,034,153	77,236	7.47	23,829	14,885	62.5
2006	1,085,100	91,375	8.42	3,544	2,830	79.9	1,064,475	79,162	7.44	24,169	15,043	62.2
2007	1,082,989	91,673	8.46	3,418	2,715	79.4	1,062,708	79,508	7.48	23,699	14,880	62.8
2008	1,085,350	90,358	8.33	3,369	2,693	79.9	1,066,645	79,075	7.41	22,074	13,976	63.3
2009	1,064,611	87,683	8.24	3,232	2,516	77.8	1,047,220	77,156	7.37	20,623	13,043	63.2
2010	1,066,636	87,638	8.22	3,238	2,553	78.8	1,049,890	77,568	7.39	19,984	12,623	63.2
2011	1,046,133	85,002	8.13	3,098	2,448	79.0	1,029,938	75,434	7.32	19,293	12,016	62.3
2012	1,033,124	83,690	8.10	2,969	2,376	80.0	1,016,354	74,091	7.29	19,739	11,975	60.7
2013	1,025,624	82,851	8.08	2,771	2,227	80.4	1,008,676	73,217	7.26	19,719	11,861	60.2
2014	999,863	79,647	7.97	2,695	2,170	80.5	983,383	70,644	7.18	19,175	11,173	58.3
2015	1,001,981	78,624	7.85	2,660	2,147	80.7	985,512	69,827	7.09	19,129	10,944	57.2
2016	973,683	75,622	7.77	2,539	2,029	79.9	957,154	67,004	7.00	19,068	10,647	55.8
2017	942,599	73,168	7.76	2,379	1,899	79.8	926,235	64,532	6.97	18,743	10,535	56.2
2018	914,970	69,968	7.65	2,192	1,738	79.3	898,732	61,248	6.81	18,430	10,458	56.7
2019	862,405	65,705	7.62	2,198	1,749	79.6	847,442	57,933	6.84	17,161	9,521	55.5
2020	838,100	61,987	7.40	1,925	1,506	78.2	823,119	54,092	6.57	16,906	9,401	55.6
2021	808,836	60,938	7.53	1,910	1,563	81.8	793,726	52,877	6.66	17,020	9,624	56.5

SVNs, small vulnerable newborns.

## DISCUSSION

This study examined secular trends in the prevalence of SVNs in the Japanese population from 1997 to 2021, using birth and stillbirth registration data from the vital statistics data. The global prevalence data of SVNs remain limited, largely due to the novelty of the SVN category and the limited availability of national data on SGA prevalence, which is available in only eight countries.^5^ However, the UNICEF–WHO group estimated the prevalence of total SVNs and their three types in 2020 as follows (per 100 livebirths): globally, the prevalence of total SVNs was 26.2, while in the region of North America, Australia, and New Zealand, Central Asia, and Europe, it was 13.8. Type-specific estimates were 8.8 globally and 7.2 regionally for preterm non-SGA, 16.3 globally and 5.9 regionally for term SGA, and 1.1 globally and 0.7 regionally for preterm SGA. Regional differences in the prevalence of preterm SVNs were small compared with those in term SGA. In Japan, the prevalence of SVNs and the three types were notably lower than the estimates by the UNICEF–WHO group, particularly for term SGA.

This study revealed that the decline in the prevalence of SVNs since 2005 is mainly attributable to a reduction in term SGA births, which carries the lowest relative risk for adverse outcomes among the three SVN subgroups. In contrast, the prevalence of preterm SGA and preterm non-SGA births—both associated with higher risk—remained essentially unchanged. This highlights the urgent need to identify modifiable risk factors, enhance risk stratification, implement proven interventions, and explore potential strategies to reduce preterm SVNs. In this study, we did not examine the underlying factors contributing to reducing the prevalence of term SGA. However, given that significant changes were observed exclusively in term SGA and based on the timing of these changes, it is plausible that subgroup-specific factors may have played a key role, rather than broad public health efforts, such as anti-smoking campaigns, pre-pregnancy underweight prevention, or gestational weight management. Additionally, we visualized regional differences in the prevalence of SVNs and their subgroups at the prefectural level. There were no significant correlations between SVN prevalence and the distribution of medical resources. Further research is needed to clarify the underlying cause of these regional disparities and to develop targeted strategies to reduce them.

Several evidence-based interventions have been identified to reduce the incidence of preterm birth. When implementing pharmacological interventions in clinical practice, it is essential to consider each country’s health insurance systems and coverage policies. Progestogen therapy has demonstrated efficacy in reducing preterm birth among women with singleton pregnancies who have either a history of spontaneous preterm birth or a short cervix.^30^ Similarly, the use of low-dose aspirin in high-risk pregnancies has been shown to significantly reduce the incidence of severe preeclampsia, thereby contributing to the prevention of preterm birth.^31^ The use of antibiotics, either therapeutically or prophylactically, for bacterial vaginosis has not been associated with a significant reduction in preterm birth overall.^32^ However, a subgroup analysis suggests a potential benefit in women with a history of preterm birth who are diagnosed with bacterial vaginosis during the current pregnancy, whereas no effect was observed in similar women without bacterial vaginosis.^33^ Emerging evidence suggests that the use of probiotics during pregnancy may be associated with increased gestational age at delivery, although findings remain inconclusive and warrant further investigation.^34^ Periodontal treatment during pregnancy has not consistently demonstrated a reduction in preterm birth, despite some studies reporting benefits in other adverse pregnancy outcomes.^35^^,^^36^ Additionally, periconceptional folic acid supplementation and food fortification have been associated with a reduced risk of preterm birth.^7^^,^^37^

In the Japanese context, where a substantial proportion of women are underweight before pregnancy, individualized nutritional counseling, including guidance on appropriate gestational weight gain, may be particularly important for reducing the risk of preterm and SGA birth.^18^^,^^21^ Reducing the risk of multiple pregnancies associated with assisted reproductive technology may help to prevent premature births, so it is necessary to comply strictly with the single-embryo transfer policy launched in 2007 by the Ethics Committee of the Japan Society for Reproductive Medicine.^38^^,^^39^ The rising incidence of syphilis in Japan since 2010 highlights the increasing importance of routine antenatal screening and early treatment to prevent congenital transmission and associated adverse outcomes, including preterm birth.^7^^,^^40^^,^^41^

In our dataset, the proportion of SVNs among LBW infants was 61.5% in 2000, 58.9% in 2010, and 56.6% in 2020. The observed discrepancy from the 99.5% reported in the previous report^2^ can be mainly attributed to differences in the threshold used to define SGA births. In Japan, the mean birth weight is lower than that in many countries, which affects the gestational age at which birth weight first falls below 2,500 g for the definition of SGA births. For example, in the INTERGROWTH-21st international reference,^42^ this occurs at 37+3 weeks for boys, aligning closely with the threshold for preterm births (below 37+0 weeks). In contrast, in the Japanese reference, it occurs later, at 38+5 weeks for primiparous deliveries and 38+0 weeks for non-primiparous deliveries. As a result, Japan has a higher proportion of non-preterm non-SGA infants with LBW, leading to a lower proportion of SVNs among LBW infants.

### Limitations

This study has two limitations. First, the accuracy of identifying SGA births may be affected by data constraints. Precise classification of SGA typically requires birth length measurements to the nearest tenth of a centimeter (eg, 50.0 cm); however, the national census data used in this study reports length only in whole centimeters (eg, 50 cm). In addition, weight and length measurements for stillbirths may be less reliable due to postmortem physiological changes occurring in utero. Accurate determination of gestational age in stillbirths can also be challenging. Nonetheless, the data quality is adequate for examining secular trends at the population level. Second, the definition of SGA in this study is based on population-specific reference values derived from Japanese birth data. The INTERGROWTH-21st Consortium has proposed international standards for birth size based on data from healthy pregnancies across eight countries under optimal conditions. However, the applicability of the standards to the Japanese population remains uncertain. In addition, these standards are designed for use from 24+0 to 42+6 weeks of gestation. In Japan, deliveries are typically induced by 41+6 weeks to avoid post-term births (≥42+0 weeks), making births beyond 42 weeks very rare. For these reasons, we employed the Japanese reference^13^ to define SGA status in this study.

### Conclusions

This study comprehensively assessed secular trends in SVNs in the Japanese population. While the overall prevalence of SVNs declined since its peak in 2005, this reduction was largely attributable to a decrease in term SGA births, with little change observed in preterm subgroups. The findings highlight persistent public health challenges related to preterm birth and underscore the need for targeted interventions.

## SUPPLEMENTARY MATERIAL

eTable 1. Distribution of gestational days (0/7∼6/7) across two time periods

eTable 2. Differences in the prevalence of SVNs from 2017 to 2021 between the national average and each prefecture, and the distribution of medical resources (N = 4,362,627)

eTable 3. Annual trends in the number and proportion of the three SVN subgroups among livebirths and stillbirths

eTable 4. Annual trends in the number and proportion of the three SVN subgroups among singleton and multiple births
