# Supplementary material for: Secular Trends in the Prevalence of Small Vulnerable Newborns in Japan, 1997–2021
Source: J Epidemiol. 2026 Mar 5;36(3):87–93. doi: 10.2188/jea.JE20240447 (PMC12893843; doi:10.2188/jea.JE20240447)
Supplement: Supplementary file 1 [file je-36-087-s001.pdf]

**eTable 1.** Distribution of gestational days (0/7~6/7) across two time periods

| Gestational days | Frequency (%) | | |
|---|---|---|---|
| | Total (period A + B)<br>1995−2021 | Period A<br>1995 and 1996 | Period B<br>1997−2021 |
| | N=29,531,752 | N=2,435,960 | N=27,095,792 |
| 0/7 | 13.6 | **0.3** | 14.8 |
| 1/7 | 14.8 | 14.7 | 14.8 |
| 2/7 | 14.4 | 14.4 | 14.4 |
| 3/7 | 14.2 | 14.2 | 14.2 |
| 4/7 | 14.0 | 14.1 | 14.0 |
| 5/7 | 13.8 | 13.9 | 13.8 |
| 6/7 | 13.7 | 13.8 | 13.7 |
| not described | **1.6** | 14.6 | **0.4** |

**eTable 2.** Differences in the prevalence of SVNs from 2017 to 2021 between the national average and each prefecture, and the distribution of medical resources (N=4,362,627)

| Prefecture | Differences between the national average and each prefecture | | | | Number of perinatal medical center (2017) | Land area per a perinatal medical center (km$^2$) | Population size per a perinatal medical center |
|---|---|---|---|---|---|---|---|
| | Overall SVN | Preterm SGA | Term SGA | Preterm non-SGA | | | |
| Hokkaido | 0.3 | 0.0 | 0.0 | 0.2 | 36 | 2,317 | 145,894 |
| Aomori | -1.0 | 0.0 | -0.5 | -0.5 | 5 | 1,929 | 249,862 |
| Iwate | 1.0 | 0.1 | 0.4 | 0.6 | 9 | 1,697 | 136,190 |
| Miyagi | -0.3 | 0.0 | -0.7 | 0.4 | 9 | 809 | 256,232 |
| Akita | 0.6 | 0.0 | 0.0 | 0.5 | 4 | 2,909 | 242,322 |
| Yamagata | -0.3 | 0.0 | -0.3 | -0.1 | 4 | 2,331 | 269,554 |
| Fukushima | -0.4 | 0.0 | 0.0 | -0.4 | 5 | 2,757 | 369,644 |
| Ibaraki | 0.5 | 0.1 | 0.3 | 0.1 | 7 | 871 | 410,479 |
| Tochigi | 1.1 | 0.0 | 0.4 | 0.8 | 8 | 801 | 242,798 |
| Gunma | -0.2 | 0.0 | -0.1 | -0.1 | 8 | 795 | 242,773 |
| Saitama | 0.2 | 0.0 | -0.1 | 0.3 | 11 | 345 | 666,423 |
| Chiba | -0.5 | 0.0 | -0.1 | -0.3 | 12 | 430 | 522,809 |
| Tokyo | -0.6 | 0.0 | -0.2 | -0.4 | 26 | 84 | 535,294 |
| Kanagawa | -0.5 | 0.0 | -0.3 | -0.2 | 20 | 121 | 460,149 |
| Niigata | 0.3 | 0.1 | -0.2 | 0.5 | 8 | 1,573 | 277,783 |
| Toyama | 0.2 | 0.0 | 0.3 | -0.1 | 6 | 708 | 173,645 |
| Ishikawa | -0.7 | 0.0 | -0.4 | -0.2 | 4 | 1,047 | 284,263 |
| Fukui | 0.2 | 0.0 | 0.4 | -0.2 | 7 | 599 | 109,911 |
| Yamanashi | 0.3 | 0.1 | 0.1 | 0.2 | 6 | 744 | 135,645 |
| Nagano | -0.3 | 0.1 | 0.4 | -0.8 | 10 | 1,356 | 205,393 |
| Gifu | 0.0 | 0.0 | -0.4 | 0.4 | 4 | 2,655 | 496,912 |
| Shizuoka | 0.0 | 0.0 | -0.3 | 0.3 | 13 | 598 | 280,135 |
| Aichi | -0.2 | 0.0 | -0.6 | 0.4 | 18 | 287 | 418,638 |
| Mie | 0.4 | 0.0 | -0.1 | 0.5 | 5 | 1,155 | 355,788 |
| Shiga | 0.4 | 0.0 | 0.1 | 0.3 | 4 | 1,004 | 353,188 |
| Kyoto | 0.7 | 0.0 | 0.4 | 0.3 | 18 | 256 | 143,485 |
| Osaka | -0.1 | 0.0 | 0.4 | -0.5 | 23 | 83 | 383,692 |
| Hyogo | 0.8 | 0.0 | 0.4 | 0.4 | 12 | 700 | 455,793 |
| Nara | 0.5 | 0.0 | 0.8 | -0.3 | 2 | 1,845 | 665,948 |
| Wakayama | 0.7 | 0.1 | 0.9 | -0.2 | 3 | 1,575 | 309,213 |
| Tottori | 1.7 | 0.2 | 1.7 | -0.3 | 2 | 1,754 | 278,338 |
| Shimane | 1.0 | 0.1 | 0.9 | 0.1 | 2 | 3,354 | 337,412 |
| Okayama | -1.1 | -0.1 | -0.1 | -0.9 | 6 | 1,186 | 315,457 |
| Hiroshima | 0.4 | 0.0 | -0.1 | 0.4 | 10 | 848 | 280,741 |
| Yamaguchi | 0.1 | -0.1 | 0.4 | -0.2 | 6 | 1,019 | 225,836 |
| Tokushima | -0.1 | -0.1 | -0.2 | 0.2 | 4 | 1,037 | 181,993 |
| Kagawa | -0.8 | -0.1 | 0.0 | -0.7 | 3 | 626 | 318,526 |
| Ehime | 0.2 | 0.1 | 0.5 | -0.4 | 6 | 946 | 223,662 |
| Kochi | 1.6 | 0.1 | 0.6 | 0.8 | 2 | 3,552 | 349,260 |
| Fukuoka | -0.3 | 0.0 | -0.1 | -0.2 | 12 | 416 | 426,509 |
| Saga | -0.7 | 0.1 | -0.3 | -0.5 | 3 | 814 | 271,607 |
| Nagasaki | 0.3 | 0.0 | 0.2 | 0.1 | 3 | 1,377 | 441,811 |
| Kumamoto | -1.1 | 0.0 | -0.8 | -0.3 | 4 | 1,852 | 436,745 |
| Oita | 0.2 | 0.0 | 0.2 | 0.1 | 4 | 1,585 | 283,341 |
| Miyazaki | 0.4 | 0.0 | 0.2 | 0.2 | 7 | 1,105 | 153,446 |
| Kagoshima | 1.6 | 0.1 | 0.5 | 1.0 | 6 | 1,531 | 266,731 |
| Okinawa | 2.7 | 0.1 | 1.3 | 1.2 | 8 | 285 | 182,052 |

SGA, small for gestational age; SVN, small vulnerable newborn.

Note: The number of perinatal medical centers by prefecture in 2017 was calculated with reference to the Ministry of Health, Labour and Welfare's list of perinatal medical centers (https://www.mhlw.go.jp/content/10800000/001238157.pdf).

**eTable 3.** Annual trends in the number and proportion of the three SVN subgroups among livebirths and stillbirths

| Year | Livebirths | | | | | | | Stillbirths | | | | | | |
|---|---|---|---|---|---|---|---|---|---|---|---|---|---|---|
| | Total | Term SGA | | Preterm non-SGA | | Preterm SGA | | Total | Term SGA | | Preterm non-SGA | | Preterm SGA | |
| | N | N | % | N | % | N | % | N | N | % | N | % | N | % |
| 1997 | 1,170,319 | 30,715 | 2.62 | 53,135 | 4.54 | 3,819 | 0.33 | 5,121 | 110 | 2.15 | 3,006 | 58.7 | 1,038 | 20.3 |
| 1998 | 1,183,911 | 32,249 | 2.72 | 54,526 | 4.61 | 4,238 | 0.36 | 5,021 | 120 | 2.39 | 2,940 | 58.6 | 1,007 | 20.1 |
| 1999 | 1,160,607 | 32,268 | 2.78 | 54,293 | 4.68 | 4,309 | 0.37 | 4,865 | 119 | 2.45 | 2,861 | 58.8 | 1,001 | 20.6 |
| 2000 | 1,175,601 | 32,472 | 2.76 | 57,431 | 4.89 | 4,230 | 0.36 | 4,666 | 106 | 2.27 | 2,742 | 58.8 | 942 | 20.2 |
| 2001 | 1,156,972 | 32,566 | 2.81 | 55,533 | 4.80 | 4,621 | 0.40 | 4,538 | 101 | 2.23 | 2,687 | 59.2 | 950 | 20.9 |
| 2002 | 1,141,106 | 33,091 | 2.90 | 55,753 | 4.89 | 4,679 | 0.41 | 4,390 | 87 | 1.98 | 2,503 | 57.0 | 906 | 20.6 |
| 2003 | 1,112,424 | 31,039 | 2.79 | 55,579 | 5.00 | 4,813 | 0.43 | 4,104 | 95 | 2.31 | 2,347 | 57.2 | 836 | 20.4 |
| 2004 | 1,101,107 | 31,443 | 2.86 | 56,382 | 5.12 | 4,865 | 0.44 | 3,885 | 79 | 2.03 | 2,276 | 58.6 | 750 | 19.3 |
| 2005 | 1,054,379 | 30,505 | 2.89 | 53,747 | 5.10 | 5,012 | 0.48 | 3,603 | 82 | 2.28 | 2,075 | 57.6 | 700 | 19.4 |
| 2006 | 1,085,100 | 30,920 | 2.85 | 55,405 | 5.11 | 5,050 | 0.47 | 3,544 | 64 | 1.81 | 2,024 | 57.1 | 742 | 20.9 |
| 2007 | 1,082,989 | 30,378 | 2.81 | 56,217 | 5.19 | 5,078 | 0.47 | 3,418 | 55 | 1.61 | 1,963 | 57.4 | 697 | 20.4 |
| 2008 | 1,085,350 | 29,226 | 2.69 | 56,185 | 5.18 | 4,947 | 0.46 | 3,369 | 79 | 2.34 | 1,937 | 57.5 | 677 | 20.1 |
| 2009 | 1,064,611 | 28,333 | 2.66 | 54,455 | 5.12 | 4,895 | 0.46 | 3,232 | 72 | 2.23 | 1,828 | 56.6 | 616 | 19.1 |
| 2010 | 1,066,636 | 27,931 | 2.62 | 54,930 | 5.15 | 4,777 | 0.45 | 3,238 | 75 | 2.32 | 1,818 | 56.1 | 660 | 20.4 |
| 2011 | 1,046,133 | 26,460 | 2.53 | 53,867 | 5.15 | 4,675 | 0.45 | 3,098 | 73 | 2.36 | 1,722 | 55.6 | 653 | 21.1 |
| 2012 | 1,033,124 | 25,839 | 2.50 | 53,259 | 5.16 | 4,592 | 0.44 | 2,969 | 41 | 1.38 | 1,697 | 57.2 | 638 | 21.5 |
| 2013 | 1,025,624 | 25,252 | 2.46 | 52,938 | 5.16 | 4,661 | 0.45 | 2,771 | 60 | 2.17 | 1,600 | 57.7 | 567 | 20.5 |
| 2014 | 999,863 | 24,365 | 2.44 | 50,814 | 5.08 | 4,468 | 0.45 | 2,695 | 47 | 1.74 | 1,549 | 57.5 | 574 | 21.3 |
| 2015 | 1,001,981 | 24,116 | 2.41 | 50,118 | 5.00 | 4,390 | 0.44 | 2,660 | 52 | 1.95 | 1,561 | 58.7 | 534 | 20.1 |
| 2016 | 973,683 | 22,681 | 2.33 | 48,791 | 5.01 | 4,150 | 0.43 | 2,539 | 45 | 1.77 | 1,488 | 58.6 | 496 | 19.5 |
| 2017 | 942,599 | 21,244 | 2.25 | 47,951 | 5.09 | 3,973 | 0.42 | 2,379 | 41 | 1.72 | 1,368 | 57.5 | 490 | 20.6 |
| 2018 | 914,970 | 19,846 | 2.17 | 46,369 | 5.07 | 3,753 | 0.41 | 2,192 | 41 | 1.87 | 1,246 | 56.8 | 451 | 20.6 |
| 2019 | 862,405 | 18,668 | 2.16 | 43,535 | 5.05 | 3,502 | 0.41 | 2,198 | 32 | 1.46 | 1,267 | 57.6 | 450 | 20.5 |
| 2020 | 838,100 | 17,315 | 2.07 | 41,216 | 4.92 | 3,456 | 0.41 | 1,925 | 26 | 1.35 | 1,101 | 57.2 | 379 | 19.7 |
| 2021 | 808,836 | 16,046 | 1.98 | 41,696 | 5.16 | 3,196 | 0.40 | 1,910 | 23 | 1.20 | 1,140 | 59.7 | 400 | 20.9 |

SGA, small for gestational age; SVN, small vulnerable newborn.

**eTable 4.** Annual trends in the number and proportion of the three SVN subgroups among singleton and multiple births

| Year | Singleton | | | | | | | Multiple births | | | | | | |
|---|---|---|---|---|---|---|---|---|---|---|---|---|---|---|
| | Total | Term SGA | | Preterm non-SGA | | Preterm SGA | | Total | Term SGA | | Preterm non-SGA | | Preterm SGA | |
| | N | N | % | N | % | N | % | N | N | % | N | % | N | % |
| 1997 | 1,154,043 | 28,832 | 2.50 | 47,554 | 4.12 | 3,592 | 0.31 | 21,397 | 1,993 | 9.31 | 8,587 | 40.1 | 1,265 | 5.91 |
| 1998 | 1,167,012 | 30,300 | 2.60 | 48,473 | 4.15 | 3,804 | 0.33 | 21,920 | 2,069 | 9.44 | 8,993 | 41.0 | 1,441 | 6.57 |
| 1999 | 1,143,153 | 30,389 | 2.66 | 47,596 | 4.16 | 3,835 | 0.34 | 22,319 | 1,998 | 8.95 | 9,558 | 42.8 | 1,475 | 6.61 |
| 2000 | 1,156,955 | 30,561 | 2.64 | 49,918 | 4.31 | 3,751 | 0.32 | 23,312 | 2,017 | 8.65 | 10,255 | 44.0 | 1,421 | 6.10 |
| 2001 | 1,138,599 | 30,784 | 2.70 | 47,957 | 4.21 | 3,961 | 0.35 | 22,911 | 1,883 | 8.22 | 10,263 | 44.8 | 1,610 | 7.03 |
| 2002 | 1,121,180 | 31,174 | 2.78 | 47,325 | 4.22 | 4,008 | 0.36 | 24,316 | 2,004 | 8.24 | 10,931 | 45.0 | 1,577 | 6.49 |
| 2003 | 1,091,984 | 29,278 | 2.68 | 46,523 | 4.26 | 4,029 | 0.37 | 24,544 | 1,856 | 7.56 | 11,403 | 46.5 | 1,620 | 6.60 |
| 2004 | 1,080,144 | 29,736 | 2.75 | 46,772 | 4.33 | 3,939 | 0.36 | 24,848 | 1,786 | 7.19 | 11,886 | 47.8 | 1,676 | 6.75 |
| 2005 | 1,034,153 | 28,817 | 2.79 | 44,371 | 4.29 | 4,048 | 0.39 | 23,829 | 1,770 | 7.43 | 11,451 | 48.1 | 1,664 | 6.98 |
| 2006 | 1,064,475 | 29,334 | 2.76 | 45,757 | 4.30 | 4,071 | 0.38 | 24,169 | 1,650 | 6.83 | 11,672 | 48.3 | 1,721 | 7.12 |
| 2007 | 1,062,708 | 28,864 | 2.72 | 46,493 | 4.37 | 4,151 | 0.39 | 23,699 | 1,569 | 6.62 | 11,687 | 49.3 | 1,624 | 6.85 |
| 2008 | 1,066,645 | 28,056 | 2.63 | 46,920 | 4.40 | 4,099 | 0.38 | 22,074 | 1,249 | 5.66 | 11,202 | 50.7 | 1,525 | 6.91 |
| 2009 | 1,047,220 | 27,065 | 2.58 | 46,018 | 4.39 | 4,073 | 0.39 | 20,623 | 1,340 | 6.50 | 10,265 | 49.8 | 1,438 | 6.97 |
| 2010 | 1,049,890 | 26,795 | 2.55 | 46,772 | 4.45 | 4,001 | 0.38 | 19,984 | 1,211 | 6.06 | 9,976 | 49.9 | 1,436 | 7.19 |
| 2011 | 1,029,938 | 25,361 | 2.46 | 46,061 | 4.47 | 4,012 | 0.39 | 19,293 | 1,172 | 6.07 | 9,528 | 49.4 | 1,316 | 6.82 |
| 2012 | 1,016,354 | 24,715 | 2.43 | 45,479 | 4.47 | 3,897 | 0.38 | 19,739 | 1,165 | 5.90 | 9,477 | 48.0 | 1,333 | 6.75 |
| 2013 | 1,008,676 | 24,071 | 2.39 | 45,195 | 4.48 | 3,951 | 0.39 | 19,719 | 1,241 | 6.29 | 9,343 | 47.4 | 1,277 | 6.48 |
| 2014 | 983,383 | 23,164 | 2.36 | 43,649 | 4.44 | 3,831 | 0.39 | 19,175 | 1,248 | 6.51 | 8,714 | 45.4 | 1,211 | 6.32 |
| 2015 | 985,512 | 22,995 | 2.33 | 43,078 | 4.37 | 3,754 | 0.38 | 19,129 | 1,173 | 6.13 | 8,601 | 45.0 | 1,170 | 6.12 |
| 2016 | 957,154 | 21,571 | 2.25 | 41,964 | 4.38 | 3,469 | 0.36 | 19,068 | 1,155 | 6.06 | 8,315 | 43.6 | 1,177 | 6.17 |
| 2017 | 926,235 | 20,151 | 2.18 | 41,051 | 4.43 | 3,330 | 0.36 | 18,743 | 1,134 | 6.05 | 8,268 | 44.1 | 1,133 | 6.04 |
| 2018 | 898,732 | 18,761 | 2.09 | 39,375 | 4.38 | 3,112 | 0.35 | 18,430 | 1,126 | 6.11 | 8,240 | 44.7 | 1,092 | 5.93 |
| 2019 | 847,442 | 17,700 | 2.09 | 37,300 | 4.40 | 2,933 | 0.35 | 17,161 | 1,000 | 5.83 | 7,502 | 43.7 | 1,019 | 5.94 |
| 2020 | 823,119 | 16,380 | 1.99 | 34,895 | 4.24 | 2,817 | 0.34 | 16,906 | 961 | 5.68 | 7,422 | 43.9 | 1,018 | 6.02 |
| 2021 | 793,726 | 15,170 | 1.91 | 35,107 | 4.42 | 2,600 | 0.33 | 17,020 | 899 | 5.28 | 7,729 | 45.4 | 996 | 5.85 |

SGA, small for gestational age; SVN, small vulnerable newborn.
